# A Strange Vision

**Published:** 1892-01

**Authors:** 


### A STRANGE VISION.

The vision of Charles XI. of Sweden, was one of the most remarkable in history. The following singular narration occurs in the Rev. J. T. James's "Travels in Sweden, Prussia and Poland." The most marvelous part of the whole affair is that, as the reader will see, no less than six persons, the monarch included, concur in attesting to the reality of this wonderful vision. Charles XI. was sitting in his chamber, between the hours of 11 and 12 at night, when he was surprised at the appearance of a light in the window of the Diet Hall. He asked Bjelke, the grand chancellor, who happened to be present, what it was he saw, and was answered that it was only the reflection of the moon. With this answer, however, he was dissatisfied, and the senator Bjelke, brother of the grand chancellor, soon entered the room, whereupon he addressed the same question to him, receiving the same answer. Soon afterward the King looked through the window and now declared that he saw persons in the Diet chamber, which was just across the street from the regal mansion. The king now rose and said: "Sirs, all is not as it should be. In the confidence that he who fears God need dread nothing, I will go and see what this may be." Ordering the two noblemen before mentioned, as also Oxenstiern and Brahe, he sent for Grunsten, the doorkeeper, and descended the staircase, making straight across the street for the Senate hall. Here the party seems to have been sensible of a certain degree of trepidation, and, no one else daring to open the door, the king took the key, unlocked it, and entered first into the ante-chamber. To their infinite surprise, it was fitted up with black cloth. Alarmed by this a second pause took place; at length the king set his foot within the hall, but fell back in astonishment at what he saw. The hall was lighted up and arrayed with the same mournful hangings as the ante-chamber; in the centre was a round table, where sat sixteen venerable men, each with large books lying open before them. Above was a king, a young man, with a crown on his head and a sceptre in his hand. At his right sat a person about forty years of age, whose face bore the strongest marks of integrity; on his left, an old man of some seventy or eighty years, who seemed very urgent with the young king that he would make a certain sign with his head, which, as often as he did, the venerable men struck their hands on their books with much violence. "Turning my eyes," says the king, "I beheld a scaffold and executioners, and men with their clothes tucked up cutting off heads so fast that the blood formed a deluge on the floor, those who suffered all seeming to be young men. Again I looked up and saw that the throne was almost overturned; near to it stood a man who seemed to be a protector of the kingdom. I trembled at these things and cried aloud: 'It is the voice of God! What ought I to understand? When shall all this come to pass?' A dead silence prevailed, but on my crying out a second time, the young king answered me, saying: 'This shall not happen in your time, but in the days of the sixth sovereign after you. He shall be of the same age which I appear now to be, and this personage sitting by me gives you the air of him who shall be protector of the realm. During the last years of the regency the country shall be sold by certain young men; and, acting in conjunction with the young king, shall establish the throne upon a sure footing, and this in such a way that never before was such a great king ever known in Sweden. All the Swedes shall be happy under him; yet before he is firmly seated on his throne an effusion of blood unparalleled in history shall take place. You have seen all; act accordingly.'" This remarkable document, the above being a literal copy, is in the Imperial Museum at Stockholm. It is signed by Charles XI., King of Sweden; H. L. Bjelke, the grand chancellor; R. Bjelke, senator; A. Oxenstiern, senator; Brahe, senator, and Petre Grunsten, Huisier, referred to in the body of the document as the door-keeper of the Diet hall. Taken all in all, it is the most wonderful vision on record, being the only one that is attested to by six persons so prominent in the world's history.—*American Notes and Queries.*

